# *Blastocystis* and *Cryptosporidium* in association with biofilms in a contaminated watercourse

**DOI:** 10.1017/S0031182025000253

**Published:** 2025-07

**Authors:** Virginia Estrada, Melisa Leone, Alicia Saura, Marisa Farber, Ludmila López-Arias

**Affiliations:** 1 Instituto de Biotecnología, Universidad Nacional de Hurlingham, Buenos Aires, Argentina; 2 Centro de Investigación y Desarrollo en Inmunología y Enfermedades Infecciosas (CIDIE-CONICET), Córdoba, Argentina; 3 Instituto de Agrobiotecnología y Biología Molecular (IABIMO), INTA-CONICET, Instituto Nacional de Tecnología Agropecuaria, Buenos Aires, Argentina

**Keywords:** Argentina, biofilm, *Blastocystis*, *Cryptosporidium*, ST3, ST8, water, watercourse

## Abstract

The objective of this study was to assess the potential role of aquatic biofilms as natural reservoirs for *Blastocystis*. For this purpose, surface water (*n* = 4) and biofilm samples (*n* = 8) were collected from a stream nearby an urban area characterized by limited sanitation infrastructure and a high prevalence of *Blastocystis* in humans. *Blastocystis* cysts were detected in three of the four water samples and seven of the eight biofilm samples using fluorescence microscopy. Furthermore, viable cysts were identified exclusively in biofilm samples (five of the eight), while no live cysts were detected in water samples. These findings indicate that aquatic biofilms provide a habitat where *Blastocystis* cysts can adhere and remain viable, potentially contributing to their environmental accumulation. In addition, molecular characterization of the five isolates identified subtypes ST8 (allele 21) and ST3 (allele 36). This study is the first to report the detection and identification of viable *Blastocystis* subtypes in aquatic biofilms. The analysis of biofilms by fluorescence microscopy, as demonstrated here, offers a promising approach for monitoring *Blastocystis* and could serve as an alternative to traditional water sampling methods.

## Graphical abstract

**Figure:**
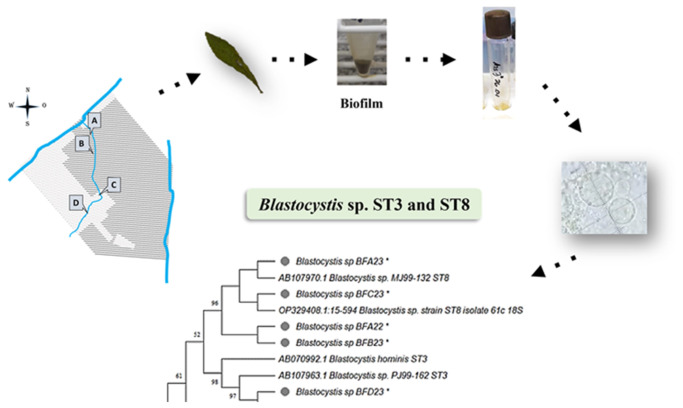

## Introduction

Water contamination by pathogens is a significant global concern, thus emphasizing the need to enhance our understanding of the primary sources of pathogens and their impacts on water resources (Pandey et al., 2014). A key gap in our knowledge of pathogenic microorganisms is understanding their survival and persistence under varying conditions and habitats (Xie et al., 2022). In aqueous environments, some microorganisms can remain suspended or adhere to organic biological particles, eventually settling to the bottom as sediment (Medema et al., 1998). Others can adhere to pre-existing biofilms – a community of microorganisms that provides protection and prevents or delays their degradation (Wingender and Flemming, 2011; Lefebvre et al., 2021).

*Blastocystis* is one of the most prevalent gastrointestinal parasites in humans (Popruk et al., 2021). Transmitted through the faecal-oral route, its cyst is considered its infective form, although its life cycle remains to be fully elucidated. The World Health Organization (WHO) has included *Blastocystis* in the ‘Water Sanitation and Health Program’ list of potential waterborne pathogens (World Health Organization, 2022). Evidence suggests that both its pathogenicity and host specificity may be linked to specific subtypes (Stensvold and Clark, 2016). To date, at least 42 subtypes (STs) have been identified from various hosts (Aykur et al., 2024). A literature review of research from 2005 to 2022 has revealed *Blastocystis* presence in water sources of 15 countries, predominantly in rivers. Eleven *Blastocystis* subtypes (ST1-ST8, ST10, ST23 and ST26) have been identified, with ST1 and ST3 being the most prevalent (Attah et al., 2023). The parasite has demonstrated the ability to survive in water for up to 1 month at 25 °C or 2 months at 4 °C (Yoshikawa et al., 2004).

The present study aimed to test the hypothesis that aquatic biofilms act as natural reservoirs for *Blastocystis*. To test this, we collected samples from upstream and downstream sections of a stream located nearby an urban area with limited sanitation infrastructure and a high prevalence of *Blastocystis* in humans (López-Arias et al., 2022).

## Materials and methods

### Study area and sample collection

**Figure 1.**
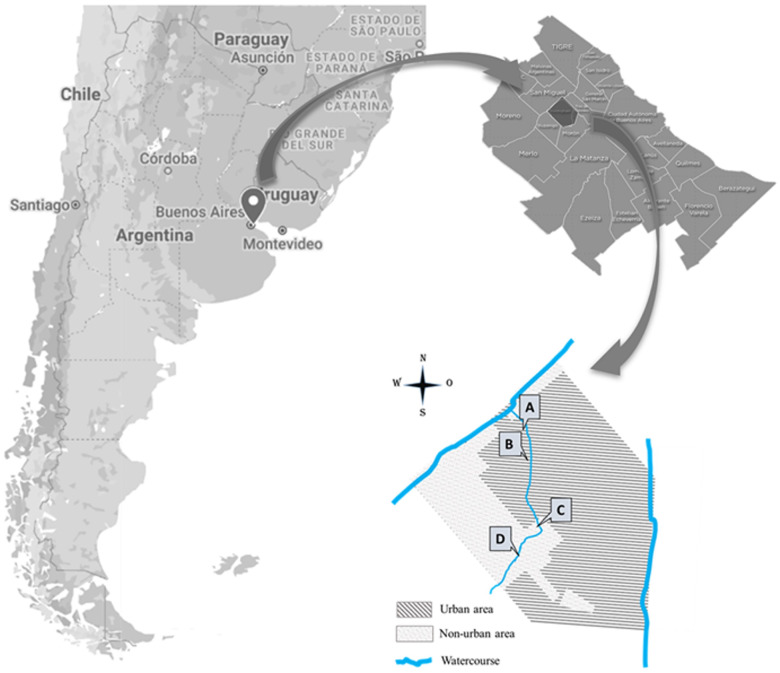
Map showing the sampling sites and watercourses in the study area.

In 2022 and 2023, environmental samples were collected from a stream in the Hurlingham district, located in the north-western region of Buenos Aires province (central-eastern Argentina). This watercourse is part of the Reconquista River Basin and flows from south to north through areas with varying degrees of urbanization and pollution from domestic and industrial wastewater. The water samples (superficial water) were collected in 5 L volumes collected from no more than 30 cm below the surface of the riverbed surface at four sites along the stream, designated A (34°34ʹ47.4″S 58°39ʹ45.4″W), B (34°35ʹ08.3″S 58°39ʹ44.1″W), C (34°36ʹ09.2″S 58°39ʹ35.6″W) and D (34°36ʹ49.5″S 58°39ʹ51.6″W) (Figure 1). Additionally, submerged plant leaves exhibiting visible biofilm formation, but no sign of decomposition, were collected in plastic tubes near the shore of the A–D sites.

### Water and biofilm processing

The surface water was decanted for 48 h, after which the sediment was concentrated by centrifugation (1500 rpm, 15 min). The supernatant was filtered through a 3 μm pore size nitrocellulose membrane (Millipore Corporation, USA) using a vacuum pump. The material retained on the filter was then combined with the sedimented material. In parallel, the biofilm was scraped from the leaves, which had been previously washed with distilled water to remove non-adherent particles. The resulting suspension was concentrated by centrifugation (1500 rpm, 15 min).

### Microscopy examination and sample culture

The concentrated water and biofilm samples were examined by optical microscope at 400× magnification for the detection of *Blastocystis*. The parasite cysts were confirmed by exposing the samples to blue light (470 nm) under a fluorescence microscope (Axio Vert A1 FL-LED Zeiss, Germany). Confirmatory studies were conducted, as other protozoan enteroparasites have been identified under the microscope. Modified Ziehl–Neelsen staining was performed on samples where oocysts compatible with Apicomplexans were detected. In cases where *Cryptosporidium* were detected, their presence was further confirmed by immunolabeling of the oocyst wall using a mouse monoclonal anti-*Cryptosporidium* oocyst antibody (dilution 1:1000; developed by Dr Saura) and a FITC-labelled polyvalent anti-mouse immunoglobulin (SIGMA, USA) was used as a secondary (dilution 1:1000).

The viability of *Blastocystis* cysts found in the samples was assessed by inoculating 200 µL of concentrated material (either water or biofilm) into tubes containing 5 mL of Jones’ medium supplemented with 5% bovine serum. The cultures were incubated at 37 °C under anaerobic conditions and examined microscopically daily. Subcultures were performed every 48–72 h.

### DNA isolation and PCR analysis

*Blastocystis* cells were harvested from cultures where growth was confirmed to extract DNA. Briefly, 250 µL of cell suspension was concentrated, resuspended in 100 µL of DNAzol (Molecular Research Center Inc., USA) and incubated for 2 h at 56 °C with 10 µL of proteinase K (TransGen Biotech Co., China). One millilitre of DNAzol was added to the sample, mixed by inversion and centrifuged at 10 000 rpm for 10 min. The supernatant was carefully transferred to another tube, and 0.75 mL of isopropanol was added. After mixing by inversion, the sample was incubated at −20 °C for 30 min and centrifuged at 12 000 rpm for 10 min. The supernatant was carefully removed, and the pellet was washed twice by adding 1 mL of 75% ethanol and mixing by inversion. The DNA pellet was dried at room temperature and resuspended in 30 µL of ultrapure water.

A PCR assay was performed in a 50 μL reaction mixture containing 0.4 μM of a primer set (Genbiotech SRL, Argentina), 0.2 mM dNTPs (TransGen Biotech Co., China), 0.5 U GoTaq DNA polymerase and 1× PCR buffer (Promega, USA) and 2 μL of DNA. The primers used were RD5_ATCTGGTTGATCCTGCCAGT and BhRDr_GAGCTTTTAACTGCAACAACG, which amplify the 600 bp barcoding region of SSU rRNA gene (Scicluna et al., 2006). The cycling conditions were 95 °C for 2 min, followed by 35 cycles at 98 °C for 30 s, 55 °C for 30 s, 72 °C for 45 s and a final extension step at 72 °C for 5 min. The amplicons were submitted to a commercial company (Macrogen, South Korea) for sequencing. Both strands were sequenced with the same primers used in the PCR.

### Phylogenetic analyses

The nucleotide sequences obtained were assembled using BioEdit software. Sequence identities were confirmed by performing alignments against the core nucleotide database from GenBank using a Basic Local Alignment Search Tool (BLASTn) (https://blast.ncbi.nlm.nih.gov/Blast.cgi). A phylogenetic analysis was conducted comparing the most frequently reported *Blastocystis* subtypes (ST1–ST10) with those isolated in this study (Jiménez et al., 2022). The analysis was performed using the maximum likelihood method and Tamura-Nei model, with bootstrap values calculated with 1000 replicates. *Proteromonas lacertae* was included as an outgroup to root the final tree. The evolutionary analysis was conducted using MEGA X (Kumar et al., 2018). Additionally, the sequences were analysed to discriminate *Blastocystis* alleles using the *Blastocystis* sp. PubMLST website: http://pubmlst.org/blastocystis/ (Jolley et al., 2018).

## Results

**Figure 2.**
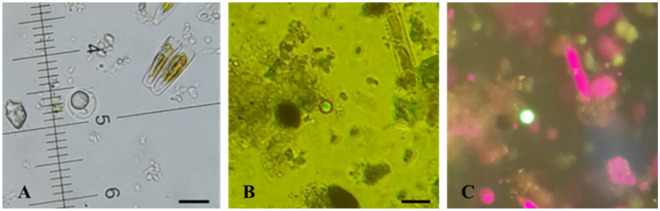
*Blastocystis* cysts detected in environmental samples under light microscopy (A) and fluorescence microscopy without exposure (B) and with blue light exposure (C). Scale bar: 10 µm.

Microscopic analysis of the samples revealed the presence of (oo)cysts of *Blastocystis, Cryptosporidium, Cyclospora* and *Giardia* in the evaluated watercourse. *Blastocystis* was further confirmed by the detection of fluorescence emitted from its cysts, which measured between 5 and 10 µm (Figure 2 and Figure S1).

The most frequently identified parasites in both in water and biofilm samples were *Blastocystis* and *Cryptosporidium*. These protists were identified in 91% and 81.8% of the samples, respectively. Both parasites were simultaneously detected in 87.5% (7/8) of the biofilm samples collected from the four sampling sites, except for site C in 2022. Conversely, *Cyclospora* and *Giardia* were present in only 18.2% and 9.1% of the samples, respectively (Table 1).

**Table 1.** Detection of enteric parasite (oo)cysts in environmental samples through microscopic examination. Presence (+); absence (−); superficial water (SW); biofilm (BF); no data (ND)

		(oo)Cyst detection by microscopic examination			
Year	ID	*Blastocystis*	*Cryptosporidium*	*Cyclospora*	*Giardia*
2022	SW A	+	+	−	−
	SW B	ND	ND	ND	ND
	SW C	+	−	+	−
	SW D	+	+	−	−
	BF A	+	+	−	−
	BF B	+	+	−	+
	BF C	−	−	−	−
	BF D	+	+	−	−
2023	BF A	+	+	−	−
	BF B	+	+	+	−
	BF C	+	+	−	−
	BF D	+	+	−	−

**Figure 3.**
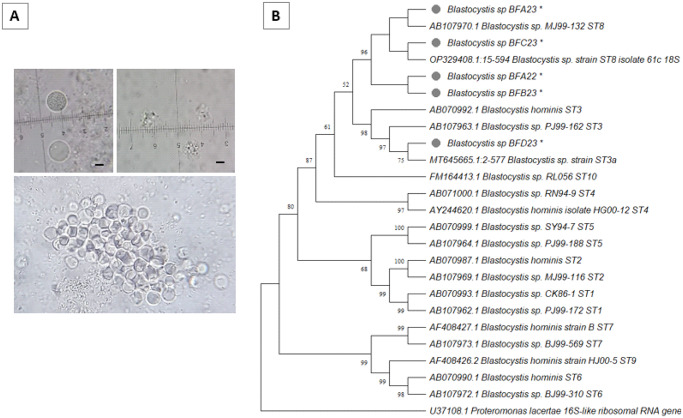
Forms of *Blastocystis* spp. observed *in vitro* xenic culture, showing aggregates of vacuolar, granular and amoeboid forms (A). Molecular phylogenetic analysis based on the maximum likelihood method. The analysis used a fragment of barcoding region of the 18S rRNA gene from different *Blastocystis* subtypes was used for the analysis. OG: *Proteromonas lacertae* (B). Note: branch values less than 50 are not shown.

Cultures confirmed the presence of viable *Blastocystis*. Specifically, no parasite growth was detected in the superficial water (SW) samples, whereas growth occurred in five out of the seven positive biofilm (BF) samples. Live cysts were present in sample BFA (2022) and all BF samples collected in 2023 (A, B, C and D). Various parasite morphologies were present, including vacuolar, granular and amoeboid forms (Figure 3A).

PCRs performed using DNA from *Blastocystis* cultures were all positives. The identification of double peaks at various sites within the chromatogram of the SSU rRNA gene from BFA (2023) indicated the potential presence of more than one subtypes. Consequently, the PCR assay and sequencing were repeated after several months of maintaining the BFA (2023) culture. The results revealed a chromatogram with unique peaks.

Phylogenetic analysis using the maximum likelihood method indicated that the isolate from sample BF D2023 exhibited the highest degree of similarity to *Blastocystis* sp. ST3, while isolates from samples BF A (2022), BF A, BF B and BF C (2023) were mostly closely related to subtype 8 (Figure 3B). Furthermore, allele 21 was identified in the isolates identified as *Blastocystis* sp. ST8, while allele 36 was found in the ST3 isolate. The nucleotide sequences of *Blastocystis* sp. obtained in this study have been submitted to GenBank (accession number PQ497564, PQ497565, PQ497566, PQ497567, PQ497568).

## Discussion

This study analysed environmental samples from an urban stream in an area with limited access to water and sewage infrastructure, alongside poor environmental sanitation. In 2019, we conducted a study on enteric parasitosis in children under 12 years old in the same area. Our findings revealed that around half of the children (57.7%) were parasitized, with protists being the most prevalent group. The seven taxa identified included *Blastocystis* spp. (26.1%), *Giardia lamblia* (13.8%), *Cryptosporidium* spp. (7.7%) and *Cyclospora cayetanensis* (1.5%), among others (López-Arias et al., 2022). In the present study, the analysed watercourse showed contamination with various parasitic protists, including *Blastocystis, Cryptosporidium, Cyclospora* and *Giardia*. Persistent sources of contamination from human activities and inadequate local sanitation conditions likely explain the presence of the same parasitic genera in both the child population and the watercourse on two separate occasions (2022 and 2023).

*Blastocystis* stood out because it was detectable in most biofilm samples collected in different sites, unlike the other parasites identified, except for *Cryptosporidium* (Table 1). This finding confirms the presence of *Blastocystis* along the watercourse. The limited literature on *Blastocystis* in sediments, particularly concerning aquatic biofilms, underscores a knowledge gap that may stem from difficulties in accurately identifying its stages in complex environmental samples. However, our fluorescence microscopy approach effectively addresses this challenge. The findings also indicated that some cysts remained viable at the time of detection. Although water samples contained cysts, cultures did not exhibit parasite growth. Conversely, most of the biofilm samples showed evidence of growth. The possibility that biofilms provide protection to *Blastocystis*, thereby prolonging its viability in the environment compared to cysts in suspension, remains unanswered and therefore requires further investigation.

Biofilms may act as environmental reservoirs, with certain pathogens attaching and therefore contributing to water pollution (Wingender and Flemming, 2011). Our results suggest that *Blastocystis* cysts adhere to aquatic biofilms and remain viable, which could lead to their accumulation in the environment. A similar phenomenon occurs with *Cryptosporidium*, whose oocysts attach to organic particles, thereby promoting sedimentation in water and adhesion to biofilms (Lefebvre et al., 2021).

In this context, our findings highlight the value of studying biofilms with fluorescence microscopy as a promising approach for epidemiological or environmental monitoring of *Blastocystis*. This method could potentially replace conventional water sampling techniques. Jellison et al. (2020) have proposed that incorporating aquatic biofilms in sampling strategies of *Cryptosporidium* could help identify high-risk regions within large, complex watersheds.

Molecular analysis of the isolates revealed that *Blastocystis* ST8 (allele 21) as the predominant subtype, detected at various sites along the stream except for site D. This subtype, uncommon in humans, has been associated with infections in animals such as rodents and pigs (Tito et al., 2019; Hublin et al., 2021). It has also been identified in both treated and untreated wastewater from treatment plants at low frequency; which suggests a degree of resistance to water treatment processes and an ability to survive in aquatic environments (Javanmard et al., 2019; Stensvold et al., 2019). In contrast, *Blastocystis* ST3 (allele 36) was present exclusively at site D. This subtype is one of the most frequent in humans (Jiménez et al., 2022), while allele 36 has been described in Spain and America in patients with gastrointestinal symptoms (Matovelle et al., 2024). In Argentina, reports of *Blastocystis* ST3 have so far included only alleles 134 and 34 (Casero et al., 2015). The double peaks observed in the chromatogram of one of the biofilm samples suggest the potential coexistence of subtypes (Guillemi et al., 2015). However, confirming this hypothesis requires further studies.

In conclusion, this study provides the first report of infectious cysts of *Blastocystis* sp. ST3 (allele 36) and ST8 (allele 21) in aquatic environmental samples from Argentina. The findings demonstrate that *Blastocystis* can adhere to aquatic biofilms and remain viable. Further research should investigate the factors influencing the sedimentation dynamics and integration of cysts into biofilms, and whether these factors enhance their survival in the environment.

## Supporting information

Estrada et al. supplementary material Estrada et al. supplementary material

## Supplementary material

The supplementary material for this article can be found at https://doi.org/10.1017/S0031182025000253.
