# Supplementary figures and images for: Blastocystis and Cryptosporidium in association with biofilms in a contaminated watercourse
Source: Parasitology. 2025 Feb 24;152(8):828–33. doi: 10.1017/S0031182025000253 (PMC12644946; doi:10.1017/S0031182025000253)

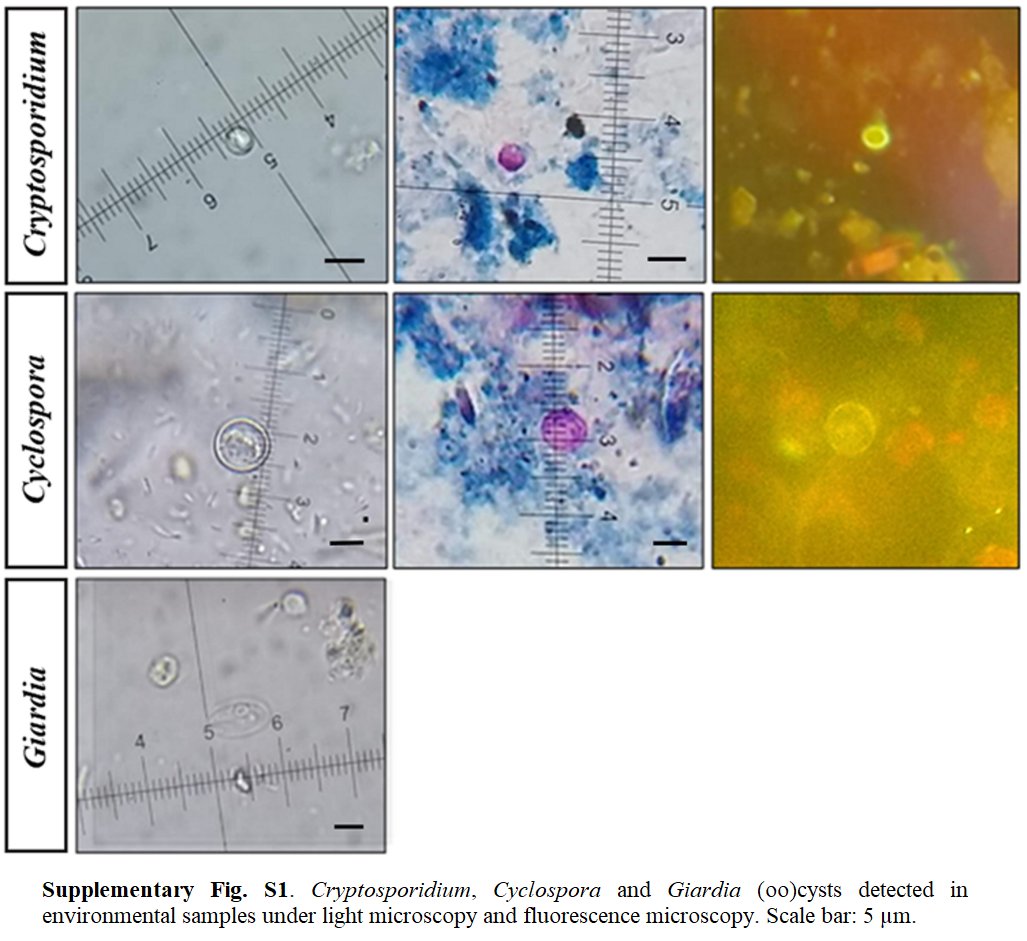

Supplement: Estrada et al. supplementary material [file S0031182025000253sup001.tif]
